# The prevalence of SCT in China, its comorbidity with ADHD and its association with life events and parental-rearing behaviors

**DOI:** 10.1038/s41598-023-43225-4

**Published:** 2023-10-07

**Authors:** Fenghua Li, Jie Luo, Yanjie Qi, Huanhuan Huang, Yuanzhen Wu, Gaoyang Xu, Zhengkui Liu, Fan He, Yi Zheng

**Affiliations:** 1 Key Lab of Mental Health, Institute of Psychology, Chinese Academy of Sciences, Beijing, China; 2 National Clinical Research Center for Mental Disorders, Beijing Key Laboratory of Mental Disorders, Beijing Anding Hospital, Beijing Institute for Brain Disorders Capital Medical University, De Sheng Men Wai An Kang Hu Tong 5 Hao, Xi Cheng Qu, Beijing, 100088 China

**Keywords:** Medical research, Epidemiology

## Abstract

Although sluggish cognitive tempo (SCT) symptoms are often observed in children with attention deficit hyperactivity disorder (ADHD), an increasing number of studies have highlighted its uniqueness. Nevertheless, no national survey on SCT among children and adolescents has been conducted in China. Hence, this research aims to study SCT in China and to evaluate the differences between SCT and ADHD symptoms by comparing their risk factors in terms of life events (LE) and parental rearing behaviors (PRB). This cross-sectional study used data from a survey on 71,929 children and adolescents in 5 province-level regions in China to study the incidence and demographic information of SCT in the Chinese population. Subsequently, the study investigated the comorbidity of ADHD and SCT, and conducted three logistic regressions on the LE and PRB scores to predict whether participants develop symptoms of ADHD or SCT, or neither symptom. 6658 participants were allocated into the SCT group, and the weighted point prevalence of SCT was 9.78%. 36.34% of participants with ADHD (n = 676) were found to demonstrate SCT symptoms, whereas no statistically significant difference was observed in its comorbidity to the three ADHD subtypes (χ^2^ = 1.668, p > 0.05, Δ = 2). The regression results on the presence or absence of ADHD revealed paternal excessive-interference and rejection, and maternal favoring were associated with ADHD diagnosis, whereas paternal punishment and favoring and maternal emotional warmth was related to the absence of ADHD symptoms. Academic stress and maternal excessive-interference were associated with SCT symptoms, and maternal emotional warmth associated with SCT absence. Concerning the presence of ADHD-only or SCT-only symptoms, LE adaptation was found to relate to SCT-only symptoms, while PRB paternal rejection and maternal favoring were associated with ADHD-only symptoms. While evidencing the high prevalence of SCT in China, our findings supported that although ADHD and SCT were highly comorbid, they may be considered two independent disorders with different risk factors. Specifically, participants with SCT symptoms are more vulnerable to stress from LE and tend to face more maladjustment than ADHD and normally-developing participants, and maternal rearing behaviours are the key factors to SCT symptoms. SCT brings global challenges in its diagnosis and treatment, and the challenge is more severe in a mentally stressful environment. Therefore, stress management and SCT etiology studies are recommended.

## Introduction

Sluggish cognitive tempo (SCT) was recognized as a sub-phenotype of attention deficit hyperactivity disorder (ADHD) inattentive subtype. However, although SCT has not yet been defined an independent disorder^1^, evidence from factor analyses of prior studies has revealed significant differences between symptoms of ADHD and SCT^2^. Specifically, a mutual variance of 25–36% was observed in ADHD-Inattentive and SCT, which implies the symptom clusters are related but not collinearly correlated^3^. Barkley reported that 59% of children with SCT were diagnosed to develop ADHD symptoms, while 39% of children with ADHD scored high in SCT symptoms^2^. Indeed, symptoms of SCT were found poorly correlated with symptoms of hyperactive-impulsive type ADHD^4^. A study demonstrated once symptoms of hyperactive-impulsive type ADHD were removed, no statistically significant correlation could be observed between ADHD and SCT^5^.

Meanwhile, existing studies have further highlighted that exclusive symptoms of SCT as lethargy, difficulty in initiating and sustaining effort, physical underactivity, excessive daydreaming, poor memory retrieval, and easy confusion or mental fogginess^6,7^, and none of them are key symptoms of ADHD. Moreover, the symptom differences between SCT and ADHD were observed via multiple measurements, such as ratings of parents and teachers, school-based behavior monitoring, clinical behavior monitoring, etc^8^. Although no study has investigated the extent to which SCT can be regarded as an independent disorder in China, studies in both Western and Eastern cultures have been establishing the cross-cultural validity of distinctive SCT symptoms^9–14^. Particularly, SCT has been studied not only in cultures rooted in individualism, but also in cultures that are similar to China in terms of the roots in Confucianism and collectivism (e.g., South Korea^10^). Notably, the distinctive characteristics of SCT were found based on both children and the self-reported assessment of senior groups, such as college students and adults, further indicating that SCT is a stable independent disorder to a greater extent^15^. Results from a recent meta-analysis demonstrated strong evidence for both internal and external validity of SCT by employing demographic information, internalization/externalization problems assessment results, cognitive dysfunctions and connections between cognition and neuropsychological functions^16^. More importantly, many studies have proffered the biological differences between SCT and ADHD. For instance, research has provided evidence that SCT symptoms are relevant to abnormal activities in the networks on orienting and attention shifting, whereas ADHD was found to be associated with abnormalities in the network on executive functions^17^. In terms of brain structures, SCT symptoms were relevant to altered frontal lobe anatomy in terms of the increased volume in terms of the anomalously large cortical regions, which indicates immaturities in functional connectivity^18,19^. This contrasted significantly with the brain anatomy and functional abnormalities of individuals developing ADHD, highlighting the distinctions between the two disorders. Taken together, it may be considered that the uniqueness of SCT has been widely evidenced by neuroimaging and heart rate variability studies^20,21^.

Beyond neurocognitive and biological explanations for SCT and ADHD, nurture factors to the development of their symptoms have received increasing attention. Particularly, although ADHD is largely considered a neurocognitive condition, factors of school and family environment have been evidenced to play significant roles (i.e., either directly or indirectly through gene-environment interactions) in whether the symptoms develop to be more impairing. Based on existing findings, life events (LE) and parental rearing behaviors (PRB) have been underlined as two environmental risk factors for ADHD^22–25^. To explain, LE refers to events related to threatening stressors or the adjustments for restoring normal life. Prior studies showed children with ADHD experienced more conflicts in LE than normally developing children^26^. As a result, the elevated level of stress imposed by LE, such as suffering from a serious illness or transferring to another school, would have a great impact on the development of children with ADHD. In PRB, earlier studies reported positive and gentle PRB correlated with improved psychological resilience children and reduced occurrence of mental disorders, including ADHD^27^. While negative and careless PRB were also found to be positively associated with increased occurrence of ADHD^28^.

Regarding SCT, twin studies have evidenced that SCT could be explained by environmental factors more prominently than neurocognitive factors, highlighting its partially distinct etiological patterns to ADHD^29^. Therefore, it is reasonable to hypothesize that LE and PRB would affect patients with ADHD and SCT differently. However, no literature to our knowledge has studied specific environmental risk factors such as LE or PRB for SCT. Based on the generally high relevance of PRB and LE to mental disorder symptoms^27^, PRB and LE may be appropriate perspectives to explore SCT. In addition, as stated above, despite the increasing cross-cultural validity and internal reliability of SCT established from samples in countries with various cultural backgrounds^9–14^, limited research has studied whether SCT can be considered independent within Chinese cultural backgrounds.

Overall, although SCT has not yet been defined as an independent disorder, existing evidence has suggested its potential independence. However, limited research has studied SCT in China. This suggests that the prevalence of SCT and the demographic information of the Chinese population developing this disorder remains largely unexplored, leaving difficulties in evaluating the importance of SCT research in China. Therefore, the first aim of the current study was (1) to investigate the prevalence and the demographic characteristics of SCT in Chinese children and adolescents by age, gender and comorbidity to the three ADHD subtypes. In addition, considering limited research has investigated the distinctions between SCT and ADHD by the environmental risk factors, we also aimed (2) to evaluate if SCT can be considered as an independent disorder in China by assessing the associations between the presence or absence of ADHD or SCT and the two primary independent variables in terms of LE and PRB.

## Materials and methods

### Study design

**Figure 1:**
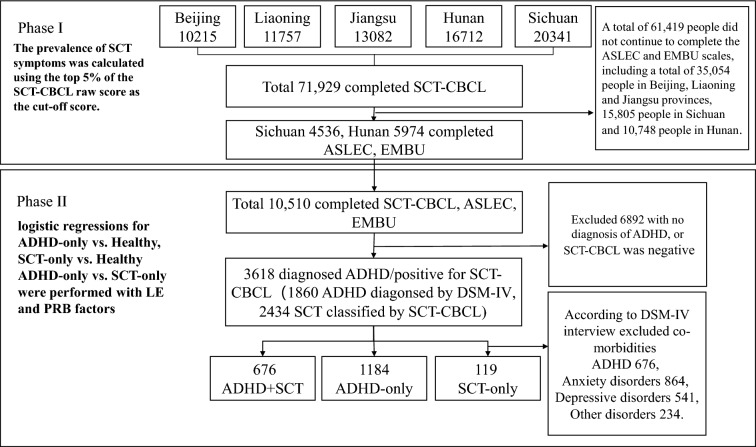
Study profile.

This study used data from a large-scale mental disorder survey for children and adolescents in China. There were two phases in this study (Fig. 1). In Phase I, researchers collected data by using the Sluggish Cognitive Tempo-Children Behavior Checklist (SCT-CBCL) from 71,929 participants aged 6–16 in 5 province-level regions (Beijing, Liaoning, Jiangsu, Hunan, Sichuan). We obtained the incidence and distribution of age and gender of SCT. In Phase II, the diagnosis of ADHD was made following the DSM-IV by two psychiatrists with at least the title of deputy chief physician. Based on the diagnoses, we investigated the comorbidity of ADHD and SCT. LE and PRB data were also collected from Hunan and Sichuan. After excluding participants developing comorbid ADHD and SCT symptoms, we allocated participants into either the ADHD-only group (i.e., participants with only ADHD symptoms), the SCT-only group (i.e., participants with only SCT symptoms) or the control group (i.e., participants who had not been diagnosed to develop any physical or psychological disorder). According to the second research aim, the dependent variable was the three groups, and the independent variables were the dimension scores of LE and PRB assessments.

To keep the homogeneity and the 1:1 sample size, participants were matched by province, age and gender in Phase II investigations on SCT and ADHD presence or absence. In matching every pair of participants, we focused on the group with a relatively small size, and all participants in the group with a relatively large size were considered as candidates to be matched. Specifically, we matched one individual from the larger group to the one participant selected from the smaller group. The selection followed a condition priority order of province, age and gender. Once a participant was selected, they would be removed from the candidate list to ensure no participant would be selected repeatedly. This process continued until we found the final pair of participants with all the conditions matched. If the matching conditions of province or gender cannot be fulfilled, the condition would be ignored, and the next condition selection would be performed in the one of the prior levels to the current subset. If the age condition is not fulfilled, individuals with the closest age would be selected for the next condition matching. All participants and their parents signed consent forms before taking part in the study. This study was approved by the Ethics Committee of Beijing Anding Hospital, Capital Medical University (2012BAI01B02).

### SCT-CBCL

SCT-CBCL (Sluggish Cognitive Tempo-Children Behavior Checklist) has been widely used in prior SCT studies. It has 4 items selected from CBCL^30^, including: 13 (confused/seems in a fog), 17 (daydreams), 80 (stares blankly) and 102 (underactive). The items were presented on a 3-point scale of 0 (not true), 1 (somewhat or sometimes true) and 2 (very true or often true). The total score ranged from 0 to 8, and larger total scores indicate more severe SCT symptoms. In our study, the Cronbach’s Alpha of this scale was 0.7.

### EMBU

EMBU (Egna Minnen Betraffande Uppfostran) is an assessment tool for PRB. The Chinese version of EMBU is a 4-point Likert scale with 66 items^31^. 58 items on 6 factors reflected the rearing behaviors of the father, including Emotional Warmth (i.e., acceptance and recognition, frequent praise, unconditional love, support and affection for the child), Severe Punishment (i.e., harsh disciplinary behavior, often through verbal or physical violence, to restrain the child's behavior), Excessive interference (i.e., controlling behavior, excessive demands on the child), Favoring (i.e., more special care for the child, usually with more parental attention and indulgence than other siblings), Rejecting (i.e., hostility, punishment, derogation and blame towards the child) and Overly-protecting (i.e., fears and anxieties, feelings of guilt and intrusions about the child's safety and health). There were also 57 items on 5 factors related to maternal rearing behaviours. The 5 factors are warm and affectionate, excessively interfering, rejecting, punishing and severe, and favoring. Participants rated 1 for “never”, 2 for “yes but seldom”, 3 for “yes, often” and 4 for “yes, always” on each item, and the score on each factor was calculated by the sum of all relevant items under it. The Cronbach’s α was between 0.717 and 0.893, and the test–retest reliability was between 0.725 and 0.871.

### ASLEC

ASLEC (Adolescents Self-Rating Life Events Checklist) was adapted from the psychological and physiological characteristics of Chinese youth in 1987 by Liu et al.^32^. There were 27 items in total, and all items were designed to measure the impact of common stress sources referring to participants’ experiences during the last 12 months. Five factors were assessed by this 5-point Likert scale, including Adaptation (change in habits, discomfort with the process of leaving or rebuilding intimate relationships), Loss (loss of finances; death of a friend or relative), Punishment (punishment and criticism at school or home), Interpersonal Relationship (being ostracized in interpersonal relationships, encountering disputes, and having unfavorable interactions with people), Academic Stress (study pressure, exam failure, strict study requirements from parents). High scores on the scales indicate more intense stress. ASLEC has been widely used in LE impact assessment in China. The Cronbach α of ASLEC is 0.91, and the comparative fit index is 0.9.

### Data analytic plan

In Phase I, we used the SCT-CBCL score at the top 5 percentile as the cutoff score^18^. Participants who scored greater than the cutoff score were considered to develop SCT symptoms. The point-weighted prevalence of SCT was also acquired. The overall sampling weights for participants in Phase I were the products of the sampling weights of each participants’ provincial region, prefectural division, county/district, school, and class. Individuals who withdrew or whose primary caregivers failed to finished the rating scale were treated as nonrespondents, and relevant adjustment was included in the poststratification process. The reciprocal of response rate in the corresponding demographic sub-group of each participant were used as their nonresponse weights^33^. SCT prevalence distributions in age spans and genders were obtained, and a chi-square test was carried out to find if the prevalence was different in males and females. In Phase II, the age difference between ADHD and SCT groups was inspected with a t-test. Comorbidity information of ADHD and SCT was acquired, and the SCT comorbidity rates in subtypes of ADHD were compared by using chi-square tests. Finally, three logistic regressions were conducted for LE and PRB scores on the presence or absence of ADHD and/or SCT symptoms. Specifically, the associations between the two primary independent variables and the presence or absence of ADHD-only symptoms, SCT-only symptoms and the presence of ADHD-only or SCT-only symptoms were assessed respectively. Dimension scores on ALSEC (punishment, loss, interpersonal relationship, academic stress, adaptation) and factor scores on EMBU (emotional warmth, severe punishment, excessive interference, favoring, rejecting and overly-protecting of father and mother) were used as independent variables, and the dependent variables were the groups. The backward step-wise method was applied in the fittings of the regression models to find the variable combinations for the best-fit models. Analyses of variance were also carried out for the regressions. All the statistics were done with the R language, version 3.5.1.

### Ethics approval and consent to participate

The project was approved by the Ethics Committee of Beijing Anding Hospital (201743FS-2) and performed in accordance with the ethical standards laid down in the 1964 Declaration of Helsinki and its later amendments. All subjects and their parents signed an informed consent form before joining the trial.

## Results

**Figure 2:**
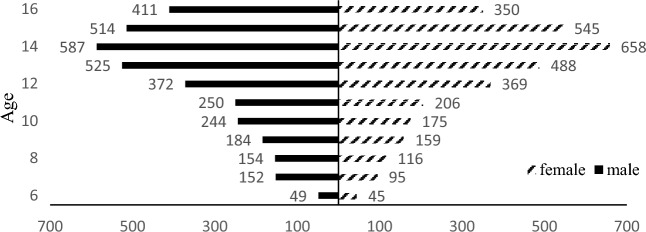
Distributions of male and female stratified by age spans.

A total of 71,929 children and adolescents aged 6–16 were recruited (*M*_Age_ = 11.48 ± 2.84), and the sample consisted of 36,430 male and 35,499 female participants. 6658 participants (*M*_*Age*_ = 12.68 ± 2.56) were allocated into the SCT group. The mean score on SCT-CBCL was found to be 0.736 (*SD* = 1.19), and the weighted point prevalence of SCT was 9.78%. Among participants in the SCT group, 3442 were male (*M*_Age_ = 12.56 ± 2.64) and 3216 were female (*M*_Age_ = 12.81 ± 2.48). There were significantly more male participants with SCT compared to female participants, *χ*^2^ = 4.047, *p* = 0.044, *Δ* = 2. The distributions of males and females stratified by age spans are presented in Fig. 2.

In Sichuan and Hunan provinces, 1860 participants (*M*_Age_ = 10.08 ± 2.81) were diagnosed to have ADHD symptoms. Among them, 551, 930 and 379 participants were diagnosed for ADHD-combined type, ADHD-Inattentive type ADHD-hyperactive-impulsive respectively. Among participants with ADHD, 676 were found to develop SCT symptoms, contributing to 36.34% of all ADHD diagnoses. In terms of the comorbidity, SCT symptoms were found in 202 participants with ADHD-combined, 339 with ADHD-inattentive and 135 with ADHD-hyperactive-impulsive symptoms. No statistically significant difference was observed in the comorbidity of SCT and the three ADHD subtypes (*χ*^2^ = 1.668, *p* > 0.05, *Δ* = 2). Based on the SCT-CBCL scale, we identified 2434 patients with SCT and the co-morbidity rate of SCT to ADHD was found to be 27.77%. Participants with SCT symptoms (*M*_Age_ = 12.83 ± 2.66) were found to be significantly older than those who developed ADHD symptoms (*M*_Age_ = 10.51 ± 2.75), *p* < 0.001.

Among participants from the two provinces, 1184 were allocated to the ADHD-only group and 119 were allocated to the SCT-only group. Table 1 shows the demographic information and comparisons for the participants in these groups and their matched control groups. Subsequently, Table 2 shows the regression results of ADHD-only and control groups. It reveals high scores on PRB factors of excessive interference and rejection from the father and favoring from the mother were associated with ADHD diagnosis; whereas low scores on punishment and favoring from the father, emotional warmth from the mother, and LE factors of interpersonal relationship were associated with ADHD diagnosis. Table 3 shows the regression results of SCT-only and control groups. It indicates high scores on the LE factor of academic stress and PRB factor of excessive-interference from the mother were associated with SCT symptoms, and low scores on the PRB factor of emotional warmth from the mother were related to SCT symptom. Table 4 depicts the regression results of ADHD-only and SCT-only. High scores on the LE factor of adaptation were observed to be associated with SCT-only symptoms, while high scores on PRB factors of rejection from the father and warmth and affection from the mother were associated with ADHD-only diagnosis.

**Table 1:** Demographic information and comparisons for the participants.

	ADHD-CON				SCT-CON				SCT-ADHD			
	ADHD	CON	*t/χ*^2^	*p*	SCT	CON	*t/χ*^2^	*p*	SCT	ADHD	*t/χ*^2^	*p*
Age (M ± SD)	10.51 ± 2.75	10.50 ± 2.31	0.086	0.931	12.83 ± 2.66	12.75 ± 2.66	0.301	0.763	12.83 ± 2.66	10.51 ± 2.75	11.26	< 0.001***
Gender (male/female)	1071/426	1077/420	0.059	0.808	104/95	103/96	0.01	0.92	104/95	1071/426	30.69	< 0.001***

Significance code: *p < 0.05, **p < 0.01, ***p < 0.001.

**Table 2:** Regression results on ADHD presence or absence

Variables	Estimate	SE	*z*	*p*
LE				
Interpersonal relationship	0.061	0.012	4.841	< 0.001***
PRB				
F-emotional warmth	0.015	0.008	1.848	0.065
F-severe punishment	− 0.021	0.009	− 2.389	0.017*
F-excessive-interference	0.061	0.015	4.054	< 0.001***
F-favoring	− 0.123	0.041	− 2.986	0.003**
F-rejection	0.039	0.019	2.017	0.044*
M-emotional warmth	− 0.028	0.008	− 3.443	< 0.001***
M-favoring	0.100	0.034	2.984	0.003**

*LE* life events, *PRB* parental rearing behaviors, *F* father, *M* mother, *SE* standard error. Significance code: *p < 0.05, **p < 0.01, ***p < 0.001.

**Table 3:** Regression results on SCT presence or absence.

Variables	Estimate	SE	*z*	*p*
LE				
Punishment	− 0.055	0.031	− 1.779	0.075
Academic stress	0.119	0.052	2.296	0.022*
PRB				
F-emotional warmth	0.030	0.020	1.489	0.137
M-emotional warmth	− 0.044	0.021	− 2.050	0.040*
M-excessive interference	0.064	0.023	2.804	0.005**
M-rejection	− 0.041	0.026	− 1.586	0.113

*LE* life events, *PRB* parental rearing behaviors, *F* father, *M* mother, *SE* standard error. Significance code: *p < 0.05, **p < 0.01, ***p < 0.001.

**Table 4:** Regression results on SCT-only presence or ADHD-only presence.

Variables	Estimate	SE	*z*	*p*
LE				
Punishment	− 0.051	0.029	− 1.772	0.076
Interpersonal relationship	− 0.068	0.042	− 1.620	0.105
Adaptation	0.091	0.037	2.444	0.015*
PRB				
F-favoring	0.152	0.078	1.953	0.051
F-rejection	− 0.061	0.028	− 2.178	0.029*
M-favoring	− 0.139	0.061	− 2.247	0.025*

*LE* life events, *PRB* parental rearing behaviors, *F* father, *M* mother, *SE* standard error. Significance code: *p < 0.05, **p < 0.01, ***p < 0.001.

## Discussion

In this study, we employed a nation-wide mental health survey dataset and investigated the prevalence and age distribution of SCT, as well as its comorbidity with ADHD symptoms. We also compared the differences between SCT and ADHD in terms of LE and PRB measured by ASLEC and EMBU with data from two provinces. The result showed that, with the score at the top 5 percentile as the cutoff score, the point prevalence of SCT was 9.78% in China. In addition, our results were in line with prior findings that although ADHD and SCT had a great overlap in participants, there are significant differences between ADHD and SCT particularly in terms of LE and PRB.

The high point prevalence of 9.78% underlined SCT as one of the most prevalent mental disorders in Chinese children and adolescents^33^. Also, 9.78% can be regarded as one of the highest point prevalence data among all surveys on SCT worldwide. Meanwhile, we found no statistically significant difference between the comorbidity rates of SCT and the three ADHD subtypes, corresponding to the result of an SCT survey conducted in the United States^7^. In addition, in line with prior studies^16^, the average age of participants with ADHD was found to be younger than that of participants with SCT in this study. Hence, our findings support that although SCT is closely related to ADHD, it demonstrates uniqueness to a greater extent. In China, SCT has not been well recognized as a distinct disorder. Instead, it has been treated as ADHD in most cases. However, no evidence has suggested that the first-line medication treatment for ADHD, such as methylphenidate, was also effective for SCT. Notably, this indicates serious problems of misdiagnosis and mistreatment under the condition of high SCT prevalence are to be solved^34,35^. Although this study was conducted in China, based on the cross-cultural validity illustrated in our introduction, we also highlight the distinction of SCT from ADHD as a problem to be raised for global mental health.

A new finding from the regression results for LE on SCT absence or presence was that greater academic stress was associated with SCT symptoms. Learning ability decrease has been proposed as inevitable for individuals with SCT^36^, and it was within our expectations to find participants with SCT being sensitive to academic stress. While Becker et al.^37^ reported lower study ability in college students was associated with SCT, one study on cognitive ability also reported that lower performance in mathematics and writing were correlated to symptoms of SCT except for daydreaming^38^ Moreover, another study conducted with college students found that SCT may be relevant to distinctive self-regulated learning strategies^39^. Taken together, our research confirms academic stress as a risk factor for the presence of SCT symptoms in Chinese children and adolescents, extending the external validity of previous findings. More importantly, the findings of the current study highlight that adaptation scores demonstrated a positive relationship to SCT exclusively for ADHD symptoms. A potential explanation for this may be that SCT is dominated by internalization rather than externalization symptoms. As previously known, maladjustment is strongly correlated with anxiety, which is often manifested as worries and fears of external uncontrollable factors. Existing studies have also revealed that SCT was more associated with anxiety and depression symptoms occurrences than ADHD^40^. Corresponding to Sevincok et al. who proposed that while the externalization symptoms were related to ADHD^41^, SCT was more associated with internalization symptoms more prominently, findings in our research provide further empirical support on the distinctions between ADHD and SCT. Results from the regression analysis on LE and ADHD presence or absence also highlighted interpersonal relationship as another risk factor for ADHD diagnosis. This corresponds to results from existing studies evidencing that children and adolescents with ADHD have a greater chance of encountering problems in social communication. Specifically, children with ADHD are more vulnerable to attacks from peers and have poorer-quality friendship^42^. On one hand, this could be related to the symptoms of ADHD, that inhibition deficiency may lead to impulsivity and overacting to unpleasant stimulations, bringing conflicts and worsening interpersonal relationships^43^. On the other hand, impairments in peer relationships also in turn lead to increases in inattention and hyperactivity or impulsivity in children with ADHD irrespective of age and gender^44^. Taken together, our findings provided further support for the vicious cycle of ADHD symptoms and poor interpersonal relationship.

Regarding PRB, both fathers’ and mothers’ parenting styles were associated with ADHD diagnosis, while only maternal parenting factors (i.e., emotional warmth and excessive interference) were associated with SCT symptoms. Furthermore, no PRB factor was observed to predict SCT diagnosis based on the logistic regression results to the SCT-only and ADHD-only groups. This indicates that mothers’ parenting behaviors are more important than that of fathers to SCT children and adolescents. Also, the results of our study highlight the lack of emotional warmth and excessive interference from mothers as critical risk factors for SCT symptoms.

Similar to many other countries and regions, father and mother play different roles in Chinese families. Specifically, previous studies have summarized the family roles in the traditional Chinese culture as “severe father and kind mother” (SFKM)^45^. This could be a possible explanation for the strong impact of fathers’ PRB on ADHD as well as its missing impact on SCT symptoms. To explain, children and adolescents with ADHD usually exhibit more externalization symptoms such as impulsivity and disobedience to the punishment and disciplines of “severe fathers”, whereas internalization symptoms demonstrated by individuals with SCT may result in fewer conflicts with parents, and so appear more relevant to the cares and interference received from “kind mothers”. The SFKM positioning may also explain why the PRB factors of favoring from father and mother were oppositely associated with ADHD diagnosis. Specifically, the differences in terms of parental role allocations may generate an observation baseline bias that favoring from the father is much rarer than that from the mother. That is, more favoring from the mother may also indicate spoiling and indulging impulsivity in the child^46^. By contrast, the association between high levels of paternal favoring and ADHD diagnosis may be explained by the usual lack of special care from fathers in Chinese culture. Another unexpected result from the logistic regression is that both favoring and punishment from the father were relevant to the absence of ADHD diagnosis. However, this association may be explained by the positivity of stringent paternal regulations in improving children’s academic performance. To elaborate, as one of the countries being categorized into the middle and low-end of the international division of labor^47^, jobs in China require more skill and knowledge to be fulfilled, whereas the payment levels remain relatively low. Indeed, this phenomenon has been further intensified by the huge size of the Chinese population. Hence, it may be implied from our results that children and adolescents in China are expected to follow strict discipline driven by punishment and rewards set by their parents, which allows them to achieve better academic outcomes in schools and colleges to secure their future.

One clinical implication based on our findings and knowledge from prior studies is that SCT patients are vulnerable to stress and tend to demonstrate maladjustment. Unlike ADHD, SCT symptoms are mostly internalizing, and this suggests that the feelings of individuals with SCT may be more easily overlooked^48^. Hence, our findings call for attention to the emotional feelings and adjustment states of children and adolescents developing SCT symptoms, particularly when they are under stressful conditions. Regarding directions for future research, the results of this study demonstrated the importance of stress as a risk factor for the developing symptoms of ADHD and SCT in multiple ways. From the perspective of etiology, long-term exposure to stress could overly activate the hypothalamic–pituitary–adrenal (HPA) axis^49^. HPA axis is a system of humoral regulation and stress response. It makes adjustments to deal with external threatening conditions by increasing adrenaline levels. This stress response helps promote adjustment level for external conditions of individuals, but consistent activation of the HPA axis is harmful to the development of nerve cells and accelerate the apoptosis process of nerve cells^50^, leading to more impairing symptoms of mental disorders^51^. As the etiological mechanisms of the different types of stress events remain unclear^52^, future studies are needed to answer this important question. In addition, a prior study reported the impact of LE on ADHD-related DNA methylation and participate in ADHD occurrence via epigenetic mechanism^53^. Concerning the commonality of ADHD and SCT, whether this process explains SCT symptoms is another important question to be studied.

Several limitations of this study should be acknowledged. Firstly, all SCT diagnoses in the current study were conducted based on the cutoff line on SCT-CBCL, whereas the SCT-CBCL was finished by families without clinician diagnosis. This may indicate a rating bias that could affect the study’s validity. However, professional diagnosis can only be available after SCT is well-recognized by the mental health community. Secondly, without prior specification of SCT, there is a possible increased type I error in statistics results. Nevertheless, findings from Phase II of our research may provide more evidence for the distinction of SCT as an independent disorder. With the current large sample size and good culture representativeness of the study, we proffer based on the findings of our study that identifying the uniqueness of SCT may not only contribute to the understanding of SCT and ADHD, but also inspire the development of their treatments. Lastly, it should be noted that Phase II of our research included participants from two rather than all five provinces being studied in Phase I to ensure a standardized procedure in LE and PRB data collection. Particularly, excluding data from the other three provinces reduced the number of participants in Phase II, and this may affect the population validity to an extent. However, the number of remaining participants was still at a satisfactory level.

## Conclusion

This study used a nation-wide mental health survey dataset to investigate the point prevalence and the demographic characteristics of individuals developing SCT symptoms in China. Specifically, we found the point prevalence of SCT in China was 9.78%. Moreover, we employed SCT-CBCL, LE and PRB survey results from two provinces, which share the participants with the national survey, to investigate the comorbidity of ADHD and SCT, as well as the associations between LE, PRB, ADHD and SCT symptoms. The findings of our study suggested that although ADHD and SCT were highly comorbid, they may be considered as two independent disorders. Particularly, our results indicated SCT patients are vulnerable to stress from LE and tend to demonstrate maladjustments. It is underlined that their emotional states need to be taken good care of, and maternal rearing behavior is the key factor. Overall, SCT brings global challenges in diagnosis and treatment, and the challenge is more severe in tight cultures such as China. Hence, we recommend further empirical research on stress management and SCT etiology.

## Data Availability

The data that support the findings of this study are available on request from the corresponding author. The data are not publicly available due to privacy or ethical restrictions.

## References

[1] Becker SP, Willcutt EG (2019). Advancing the study of sluggish cognitive tempo via DSM, RDoC, and hierarchical models of psychopathology. Eur. Child Adolesc. Psychiatry..

[2] Barkley RA (2013). Distinguishing sluggish cognitive tempo from ADHD in children and adolescents: Executive functioning, impairment, and comorbidity. J. Clin. Child Adolesc..

[3] Owens EB, Hinshaw SP, Mcburnett K, Pfiffner L (2018). Predictors of response to behavioral treatments among children with ADHD-inattentive type. J. Clin. Child Adolesc. Psychol..

[4] Hartman CA, Willcutt EG, Rhee SH, Pennington BF (2004). The relation between sluggish cognitive tempo and DSM-IV ADHD. J. Abnorm. Child Psychol..

[5] Harrington KM, Waldman ID (2010). Evaluating the utility of sluggish cognitive tempo in discriminating among DSM-IV ADHD subtypes. J. Abnorm. Child Psychol..

[6] Lee S, Burns GL, Snell J, Mcburnett K (2014). Validity of the sluggish cognitive tempo symptom dimension in children: Sluggish cognitive tempo and ADHD-inattention as distinct symptom dimensions. J. Abnorm. Child Psychol..

[7] Servera M, Sáez B, Burns GL, Becker SP (2018). Clinical differentiation of sluggish cognitive tempo and attention-deficit/hyperactivity disorder in children. J. Abnorm. Psychol..

[8] Becker SP (2021). Systematic review: Assessment of Sluggish cognitive tempo over the past decade. J. Am. Acad. Child Psychiatry..

[9] Başay Ö, Çiftçi E, Becker SP, Burns GL (2021). Validity of Sluggish cognitive tempo in Turkish children and adolescents. Child Psychiatry Hum. Dev..

[10] Lee S, Burns GL, Becker SP (2018). Toward establishing the transcultural validity of Sluggish cognitive tempo: Evidence From a sample of South Korean children. J. Clin. Child Adolesc..

[11] Takeda T, Burns GL, Jiang Y, Becker SP, Mcburnett K (2019). Psychometric properties of a sluggish cognitive tempo scale in Japanese adults with and without ADHD. Atten. Deficit Hyperact. Disord..

[12] Sadeghi-Bahmani D, Mohammadian Y, Ghasemi M (2022). Sluggish cognitive tempo among iranian children and adolescents: A validation study of the Farsi child and adolescent behavior inventory (CABI)-parent version. J. Clin. Med..

[13] Burns GL, Preszler J, Ahnach A, Servera M, Becker SP (2022). Multisource network and latent variable models of Sluggish cognitive tempo, ADHD-inattentive, and depressive symptoms with Spanish children: Equivalent findings and recommendations. Res. Child Adolesc. Psychopathol..

[14] Belmar M, Servera M, Becker SP, Burns GL (2017). Validity of Sluggish cognitive tempo in South America: An initial examination using mother and teacher ratings of chilean children. J. Atten. Disord..

[15] Becker SP, Burns GL, Smith ZR, Langberg JM (2020). Sluggish cognitive tempo in adolescents with and without ADHD: Differentiation from adolescent-reported ADHD inattention and unique associations with internalizing domains. J. Abnorm. Child Psychol..

[16] Becker SP, Leopold DR, Burns GL (2016). The internal, external, and diagnostic validity of Sluggish cognitive tempo: A meta-analysis and critical review. J. Am. Acad. Child Psychiatry..

[17] Posner J, Park C, Wang Z (2014). Connecting the dots: A review of resting connectivity MRI studies in attention-deficit/hyperactivity disorder. Neuropsychol. Rev..

[18] Camprodon-Rosanas E, Pujol J, Martínez-Vilavella G (2019). Brain structure and function in school-aged children with Sluggish cognitive tempo symptoms. J. Am. Acad. Child Psychiatry..

[19] Becker SP, Burns GL, Leopold DR, Olson RK, Willcutt EG (2018). Differential impact of trait sluggish cognitive tempo and ADHD inattention in early childhood on adolescent functioning. J. Child Psychol. Psychiatry.

[20] Sussman TJ, Posner J (2019). Editorial: Neural correlates of Sluggish cognitive tempo: Biological evidence of a distinct clinical entity?. J. Am. Acad. Child Adolesc. Psychiatry.

[21] Yung TW, Lai CY, Chan JY, Ng SS, Chan CC (2020). Neuro-physiological correlates of sluggish cognitive tempo (SCT) symptoms in school-aged children. Eur. Child Adolesc. Psychiatry.

[22] Combs MA, Canu WH, Broman-Fulks JJ, Rocheleau CA, Nieman DC (2015). Perceived stress and ADHD symptoms in adults. J. Atten. Disord..

[23] Sellers R, Harold GT, Smith AF (2021). Disentangling nature from nurture in examining the interplay between parent–child relationships, ADHD, and early academic attainment. Psychol. Med..

[24] Shelleby EC, Ogg J (2020). Longitudinal relationships between parent involvement, parental warmth, ADHD symptoms, and reading achievement. J. Atten. Disord..

[25] Humphreys KL, Watts EL, Dennis EL, King LS, Thompson PM, Gotlib IH (2019). Stressful life events, ADHD symptoms, and brain structure in early adolescence. J. Abnorm. Child Psychol..

[26] Friedrichs B, Igl W, Larsson H, Larsson JO (2012). Coexisting psychiatric problems and stressful life events in adults with symptoms of ADHD—A large Swedish population-based study of twins. J. Atten. Disord..

[27] Eun JD, Paksarian D, He JP, Merikangas KR (2018). Parenting style and mental disorders in a nationally representative sample of US adolescents. Soc. Psychiatry Psychiatr. Epidemiol..

[28] Harold GT, Leve LD, Barrett D (2013). Biological and rearing mother influences on child ADHD symptoms: Revisiting the developmental interface between nature and nurture. J. Child Psychol. Psychiatry.

[29] Moruzzi S, Rijsdijk F, Battaglia M (2014). A twin study of the relationships among inattention, hyperactivity/impulsivity and sluggish cognitive tempo problems. J. Abnorm. Child Psychol..

[30] Bilenberg N (1999). The Child Behavior Checklist (CBCL) and related material: standardization and validation in Danish population based and clinically based samples. Acta Psychiatr. Scand. Suppl..

[31] Yue DM, Li MG, Jin HH, Ding BK (1993). Preliminary revision of EMBU and its application in neurotic patients. Chin. Ment. Health..

[32] Liu, X., Liu, L. Q., Yang, J., Chai, F., Ma, D. Reliability and validity of the adolescents self-rating life events checklist. (1997).

[33] Li F, Cui Y, Li Y (2022). Prevalence of mental disorders in school children and adolescents in China: Diagnostic data from detailed clinical assessments of 17,524 individuals. J. Child Psychol. Psychiatry..

[34] Froehlich TE, Becker SP, Nick TG (2018). Sluggish cognitive tempo as a possible predictor of methylphenidate response in children with ADHD: A randomized controlled trial. J. Clin. Psychiatry..

[35] Fırat S, Gul H, Aysev A (2021). An open-label trial of methylphenidate treating Sluggish cognitive tempo, inattention, and hyperactivity/impulsivity symptoms among 6- to 12-year-old ADHD children: What are the predictors of treatment response at home and school?. J. Atten. Disord..

[36] Cook NE, Braaten EB, Vuijk PJ (2019). Slow processing speed and Sluggish cognitive tempo in pediatric attention-deficit/hyperactivity disorder: Evidence for Differentiation of functional correlates. Child Psychiatry Hum. Dev..

[37] Becker SP, Dvorsky MR, Tamm L, Willoughby MT (2021). Preschool neuropsychological predictors of school-aged Sluggish cognitive tempo and inattentive behaviors. Res. Child Adoles. Psychopathol..

[38] Tamm L, Garner AA, Loren R (2016). Slow sluggish cognitive tempo symptoms are associated with poorer academic performance in children with ADHD. Psychiatry Res..

[39] Shelton CR, Addison WE, Hartung CM (2019). ADHD and SCT symptomatology in relation to college students' use of self-regulated learning strategies. J. Atten. Disord..

[40] Hossain B, Bent S, Parenteau C, Widjaja F, Davis M, Hendren RL (2022). The associations between Sluggish cognitive tempo, internalizing symptoms, and academic performance in children with reading disorder: A longitudinal cohort study. J. Atten. Disord..

[41] Sevincok D, Ozbay HC, Ozbek MM, Tunagur MT, Aksu H (2020). ADHD symptoms in relation to internalizing and externalizing symptoms in children: The mediating role of sluggish cognitive tempo. Nord. J. Psychiatry..

[42] Rokeach A, Wiener J (2020). Friendship quality in adolescents with ADHD. J. Atten. Disord..

[43] Lee Y, Mikami AY, Owens JS (2021). Children’s ADHD symptoms and friendship patterns across a school year. Res. Child Adolesc. Psyhopathol..

[44] Tseng WL, Kawabata Y, Gau SSF (2014). Symptoms of attention-deficit/hyperactivity disorder and peer functioning: A transactional model of development. J. Abnorm. Child Psychol..

[45] Li N, Hein S (2019). Parenting, autonomy in learning, and development during adolescence in China. New Dir. Child Adolesc. Dev..

[46] Mclaughlin DP, Harrison CA (2006). Parenting practices of mothers of children with ADHD: The role of maternal and child factors. Child Adol. Ment. H-Uk..

[47] Fan CC (2003). Rural-urban migration and gender division of labor in transitional China. Int. J. Urban Reg..

[48] Becker SP, Luebbe AM, Fite PJ, Stoppelbein L, Greening L (2014). Sluggish cognitive tempo in psychiatrically hospitalized children: Factor structure and relations to internalizing symptoms, social problems, and observed behavioral dysregulation. J. Abnorm. Child Psychol..

[49] Guilliams TG, Edwards L (2010). Chronic stress and the HPA axis. Standard..

[50] Leonard BE (2005). The HPA and immune axes in stress: The involvement of the serotonergic system. Eur. Psychiatry..

[51] Wingenfeld K, Wolf OT (2011). HPA axis alterations in mental disorders: Impact on memory and its relevance for therapeutic interventions. CNS Neurosci. Ther..

[52] Essex MJ, Shirtcliff EA, Burk LR (2011). Influence of early life stress on later hypothalamic–pituitary–adrenal axis functioning and its covariation with mental health symptoms: A study of the allostatic process from childhood into adolescence. Dev. Psychopathol..

[53] Sun Y, Jia T, Barker ED (2023). Associations of DNA methylation with behavioral problems, gray matter volumes, and negative life events across adolescence: Evidence from the longitudinal IMAGEN study. Biol. Psychiatry..

